# Microbe-Friendly Plants Enable Beneficial Interactions with Soil Rhizosphere Bacteria by Lowering Their Defense Responses

**DOI:** 10.3390/plants13213065

**Published:** 2024-10-31

**Authors:** Alexander Arkhipov, Ziyu Shao, Sean R. Muirhead, Muchineripi S. Harry, Maria Batool, Hooman Mirzaee, Lilia C. Carvalhais, Peer M. Schenk

**Affiliations:** 1 Plant-Microbe Interactions Laboratory, School of Agriculture and Food Sustainability, The University of Queensland, Brisbane, QLD 4072, Australia; arkhipovalexander1@gmail.com (A.A.); ziyu.shao@uq.edu.au (Z.S.); sean.muirhead@erm.com (S.R.M.); m.harry@uqconnect.edu.au (M.S.H.); m.batool@uq.edu.au (M.B.); hooman.mirzaee@uq.net.au (H.M.); 2 Center for Horticultural Science, Queensland Alliance for Agriculture and Food Innovation, The University of Queensland, Ecosciences Precinct, Brisbane, QLD 4072, Australia; l.carvalhais@uq.edu.au; 3 Sustainable Solutions Hub, Global Sustainable Solutions Pty Ltd., Brisbane, QLD 4105, Australia; 4 Centre for Bioinnovation, The University of the Sunshine Coast, Sippy Downs, QLD 4556, Australia

**Keywords:** beneficial microbe, biostimulant, microbial biofertilizer, PGPR, plant breeding, plant defense, plant growth-promoting rhizobacteria, plant–microbe interactions, rhizosphere bacteria, sustainable agriculture

## Abstract

The use of plant growth-promoting rhizobacteria presents a promising addition to conventional mineral fertilizer use and an alternative strategy for sustainable agricultural crop production. However, genotypic variations in the plant host may result in variability of the beneficial effects from these plant–microbe interactions. This study examined growth promotion effects of commercial vegetable crop cultivars of tomato, cucumber and broccoli following application with five rhizosphere bacteria. Biochemical assays revealed that the bacterial strains used possess several nutrient acquisition traits that benefit plants, including nitrogen fixation, phosphate solubilization, biofilm formation, and indole-3-acetic acid (IAA) production. However, different host cultivars displayed genotype-specific responses from the inoculations, resulting in significant (*p* < 0.05) plant growth promotion in some cultivars but insignificant (*p* > 0.05) or no growth promotion in others. Gene expression profiling in tomato cultivars revealed that these cultivar-specific phenotypes are reflected in differential expressions of defense and nutrient acquisition genes, suggesting that plants can be categorized into “microbe-friendly” cultivars (with little or no defense responses against beneficial microbes) and “microbe-hostile” cultivars (with strong defense responses). These results validate the notion that “microbe-friendly” (positive interaction with rhizosphere microbes) should be considered an important trait in breeding programs when developing new cultivars which could result in improved crop yields.

## 1. Introduction

Modern crop productivity and pest control rely heavily on chemical fertilizers and pesticides, which can have harmful effects on human health and the environment [1,2,3]. The use of plant growth-promoting rhizobacteria (PGPR) for crop growth and yield promotion is an emerging alternative to chemical fertilizer use for sustainable production systems that now extends to modern industrialized food production systems [3,4,5]. The use of PGPR for future sustainable agricultural crop production is promising with its relatively low impact on native soil microorganisms, livestock and humans, and minimal impacts on soil ecology and biodiversity [3,6,7].

PGPR are able to influence the growth of plants through the uptake of nutrients, production of phytohormones and improving tolerance to abiotic stresses [8,9]. Bioavailable nutrients are provided by PGPR through their microbial biomass and their ability to fix atmospheric nitrogen, solubilize phosphorous, and access minerals (e.g., Fe through siderophores). Some PGPR metabolize the ethylene precursor 1-aminocyclopropane-1-carboxylate (ACC), resulting in reduced abiotic stress, or produce biostimulants and plant hormones, such as indole acetic acid (auxin). In addition, PGPR can control pathogens through priming, leading to induced systemic resistance (ISR) defense responses in plants, direct antagonism through the production of antimicrobial compounds, or by competing against pathogens [8,9]. Plant growth and yield greatly depend on the availability of nutrients in the soil, particularly at the soil–root interface, which is influenced by many factors, including climate, edaphic factors and soil type, plant genotype, soil microorganisms, etc. [10,11,12]. Microorganisms, especially PGPR, can aid their plant hosts to acquire nutrients by influencing their bioavailability (e.g., N fixation, P solubilization, Fe chelation, etc.) in the rhizosphere and/or influencing the main mechanisms involved in the nutritional process [10,11,13,14]. In addition, the microbial biomass in the rhizosphere provides a rich source of organic nutrients when microbial cells lyse or are being degraded [15,16].

There are, however, numerous challenges that must be addressed in this field of research, including inconsistencies between different studies usually caused by differences in climatic/edaphic conditions, and positive results observed in greenhouse trials can fail to succeed in field trials [17]. In addition, genotypic and phenotypic variations in plants may result in variability of beneficial effects from the plant–microbe interactions [18,19]. The latter authors reported that the natural variation in *Arabidopsis* plant genotypes dictates how much the plant benefits from the PGPR isolates, i.e., the plant genetic variation is related to the capacity of plant hosts to profit from plant–microbe interactions [19].

Plant genotype can determine the composition of the rhizosphere microbial consortia structures which may benefit plant growth [20,21,22]. For example, different host *Arabidopsis* plant biovars can specifically select and attract the specific types and number of beneficial bacteria species (e.g., Pseudomonaceae members) by producing different anti-microbial compounds [23]. Similarly, Pérez-Jaramillo et al. [22] compared eight common bean accessions and demonstrated that their genotypes determined the assembly of their rhizosphere microbiome with only 0.7% operational taxonomic units (OTUs) being shared between these accessions. This kind of genotype-involved selection does not only affect growth-promoting interactions, but it also affects bacteria-induced plant defense responses [23,24].

Cultivar-specific responses have been observed in several economically important crop plants, including wheat, rice, maize and tomato to PGPR isolates from different genera, such as *Azospirilum* sp., *Bacillus* sp. and *Pseudomonas* sp. [25,26,27,28,29,30,31]. Khalid et al. [28] and Chanway et al. [25] showed cultivar-specific responses of wheat to different *Bacillus* isolates and unidentified species of PGPR. Similarly, Rozier et al. [29] reported wheat cultivar-specific responses to *Azospirillum lipoferum* that resulted in the accelerated radicle emergence of one cultivar and a delayed upgrading of seedling defenses. Drogue et al. [27] and Sasaki et al. [30] reported that the genotype of rice affected the plant’s response to inoculation with *Azospirillum* sp. isolates. Delfin et al. [26] tested the response of ten tomato cultivars under field conditions to the commercial PGPR product BioGroTM composed of *Pseudomonas fluorescens*/*putida*, *Klebsiella pneumoniae* and *Citrobacter freundii* [31]. The authors reported that half of the cultivars responded positively, while the other half responded in a negative manner (i.e., reduced shoot dry weight [26]), providing further evidence that the plant genotype plays a role in the nature of interactions with microbes. Similarly, certain tomato recombinant inbred lines under drought stress also performed better with microbial inoculants than others [32].

In the past, it was believed that soil types and plant species were the main factors that influenced the microbial community composition, which would influence plant health and growth rates [33]. Other studies have shown that in long-term agricultural systems or in natural environmental associates conducive to plant–microbe co-evolution, plant host genotypes play a key role in shaping host–environment and host–symbiosis interactions, which act as natural selection [23]. We recently hypothesized that different plant defense responses from “microbe-friendly” and “microbe-hostile” genotypes could explain the outcome for plant growth from PGPR–plant interactions [34].

Microbe-friendly plant cultivars could be more capable of recruiting and accommodating beneficial PGPR depending on their energy investment in, for example, either growth promotion or disease control [4,19]. Alternatively, microbe-hostile plant cultivars may have lost some of their ability to recruit and/or accommodate beneficial PGPR isolates due to modern plant breeding which may have excluded genetic traits in plant hosts that are essential for interacting and hosting beneficial microorganisms [18,33,35].

Modern plant breeding programs typically utilize mono-cropping systems under optimal conditions which lead to a reduction in crop biodiversity and diminish the contribution of the rhizosphere microbiome to plant growth and health [33]. Moreover, they may have selected against genetic traits in plant hosts that are essential for interacting and hosting beneficial microorganisms [33]. Over the past few decades, tomato plant breeding has resulted in a loss in genetic diversity, and it has been suggested that modern breeding programs should focus on reintroducing the desirable traits from wild tomato species [36].

To test the compatibility of plant genotype and beneficial rhizosphere bacteria, the current study assessed four PGPR strains as biofertilizers on three commercial crops (seven cultivars), including broccoli, cucumber and tomato. Indeed, different host plant cultivars responded differently (i.e., positively or negatively) and perceived the beneficial PGPR isolates as either microbe-friendly or microbe-hostile. Marker gene expression analyses suggest that the ability for plants to interact with PGPR in a beneficial manner relies on plants having lower basal defense levels and being able to suppress defense responses.

## 2. Results

### 2.1. Identification of Bacterial Isolates and Plant Beneficial Traits

Using 16S rDNA amplicon sequencing identified the four bacterial isolates used in this study, namely, 33YE: *Bacillus amyloliquefaciens*, UQ2077A: *Enterobacter ludwigii*, UQ4510An: *Pseudomonas azotoformans* and UQ9000N: *Bacillus velezensis* (Table 1).

The phylogenetic trees for each of the newly isolated UQ2077A, UQ4510An and UQ9000N strains can be seen in Supplementary Figure S1. Using in vitro assays for various plant growth promotion traits, it was found that isolates 33YE and UQ2077A were able to produce IAA and biofilm, fix atmospheric nitrogen and solubilize phosphate. Similarly, both UQ4510An and UQ9000N were able to produce IAA and biofilm and fix nitrogen; however, they were unable to solubilize phosphate based on the plate assays used.

### 2.2. Broccoli Pot Trials

**Figure 1:**
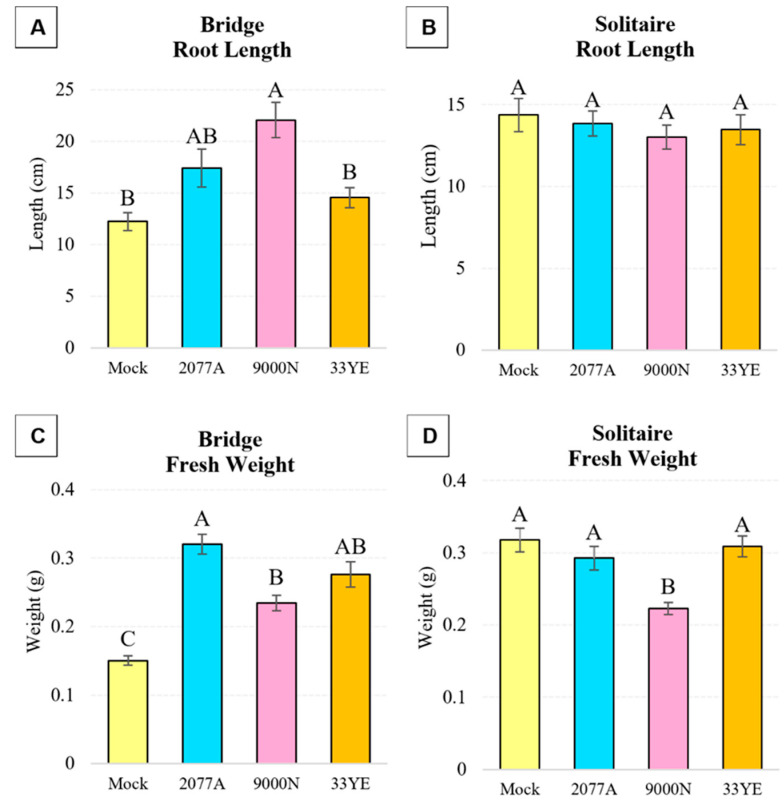
Phenotypic analysis of broccoli plants (*B. oleracea* var. *italica*; cv. Bridge and Solitaire) after treatment with *E. ludwigii* UQ2077A, *B. velezensis* UQ9000N, *B. amyloliquefaciens* 33YE and mock-treatment with 1xPBS (Mock) as control. Shown are mean values ± SE (*n* = 30 plants per treatment) of (**A**,**B**) root length and (**C**,**D**) fresh weight of 7-week-old plants (4 weeks after bacterial inoculation). Statistical significance was determined via ANOVA and Tukey’s HSD. If the letters A–C are not shared between different treatments, this indicates a statistically significant difference (*p* ≤ 0.05).

Broccoli plants, or cultivar Bridge, responded positively to the root inoculation by PGPR isolates with a significant (*p* < 0.05) increase of 181% following UQ9000N, and non-significant (*p* > 0.05) positive trends of 143% and 119% after treatment with UQ2077A and 33YE, respectively, compared with mock-treated control plants (Figure 1A). Similarly, the fresh weight of cultivar Bridge significantly (*p* < 0.05) increased by 213%, 156% and 184% by UQ2077A, UQ9000N and 33YE treatments, respectively (Figure 1C). On the other hand, for cultivar Solitaire, root length and fresh weight did not significantly (*p* > 0.05) increase by treatment with any of the three isolates, and UQ9000N even displayed a significantly (*p* < 0.05) lower fresh weight by 30% (Figure 1B,D).

### 2.3. Cucumber Pot Trials

**Figure 2:**
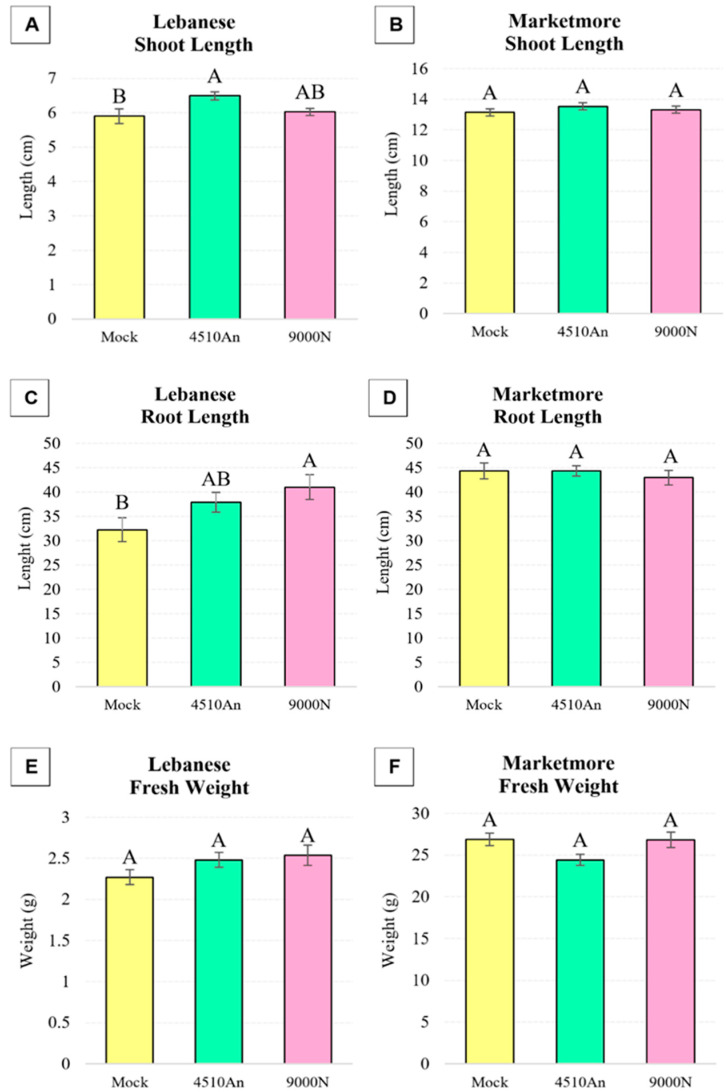
Phenotypic analysis of cucumber plants (C. sativus; cv. Lebanese and Marketmore) after treatment with *P. azotoformans* UQ4510An, *B. velezensis* UQ9000N and mock-treatment with 1xPBS (Mock) as control. Shown are mean values ± SE (n = 30 plants per treatment) of (**A**,**B**) shoot length, (**C**,**D**) root length and (**E**,**F**) fresh weight of 3-week-old plants (1 week after bacterial inoculation). Statistical significance was determined via ANOVA and Tukey’s HSD. If the letters A–B are not shared between different treatments, this indicates a statistically significant difference (*p* ≤ 0.05).

Two cultivars of cucumber plants, Lebanese and Marketmore, were grown for 3 weeks with one root treatment at 2 weeks of growth with two PGPR isolates, *P. azotoformans* UQ4510An and *B. velezensis* UQ9000N. It was found that these two cucumber cultivars reacted differently to the bacterial treatments (Figure 2). UQ4510An significantly (*p* < 0.05) increased the shoot length of the Lebanese cultivar by 110% compared with mock-treated control plants, while UQ9000N significantly (*p* < 0.05) increased the root length by 127%, and fresh weight was not significantly affected by either of the isolates compared with the control (Figure 2A,C,E). However, none of the three phenotypic parameters of the Marketmore cultivar were significantly (*p* > 0.05) affected by treatments with either UQ4510An or UQ9000N compared with the control (Figure 2B,D,F).

### 2.4. Tomato Pot Trials

**Figure 3:**
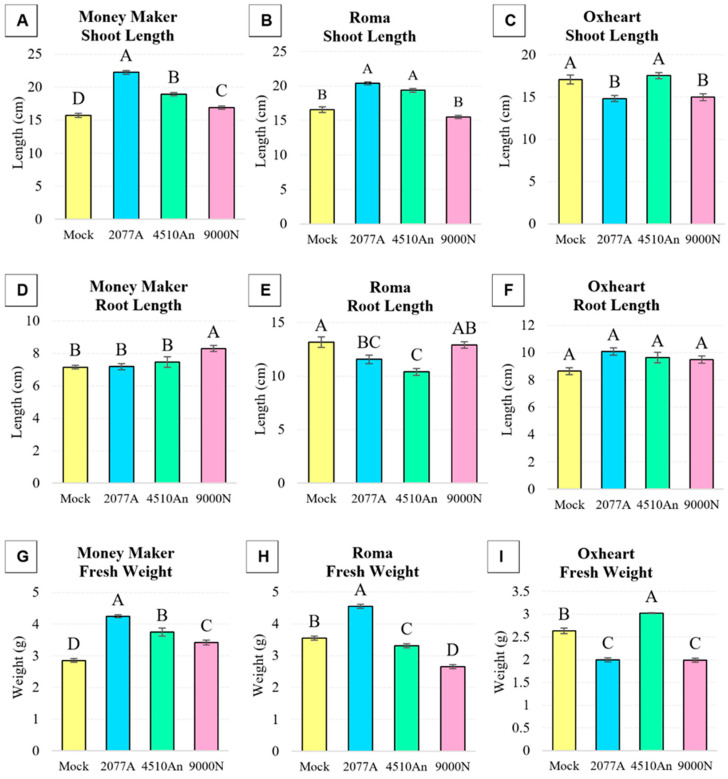
Phenotypic analysis of tomato plants (*S. lycopersicum*; cv. Money Maker, Roma and Oxheart) after treatment with *E. ludwigii* UQ2077A, *P. azotoformans* UQ4510An*, B. velezensis* UQ9000N or 1xPBS (Mock) as a control. Shown are mean values ± SE (n = 30 plants per treatment) of (**A**–**C**) shoot length, (**D**–**F**) root length and (**G**–**I**) fresh weight of 3-week-old plants (1 week after bacterial inoculation). Statistical significance was determined via ANOVA and Tukey’s HSD. If the letters A–D are not shared between different treatments, this indicates a statistically significant difference (*p* ≤ 0.05).

Three cultivars of tomato plants, including Money Maker, Roma and Oxheart were grown for 3 weeks with one root treatment at 2 weeks of growth with three PGPR isolates, namely *E. ludwigii* UQ2077A, *P. azotoformans* UQ4510An and *B. velezensis* UQ9000N. As with previous crop plants, different cultivars responded differently to the bacterial treatments (Figure 3). All of the three PGPR isolates positively influenced the growth of Money Maker which significantly (*p* < 0.05) increased shoot length and fresh weight by 142% and 149%, respectively, for UQ2077A, 120% and 131%, respectively, for UQ4510An, and 107% and 120%, respectively, and for UQ9000N treatments compared with the mock-treated control (Figure 3A,G). Only UQ9000N significantly (*p* < 0.05) increased the root length of Money Maker by 115% compared with the control (Figure 3D). On the other hand, UQ2077A and UQ4510An significantly increased the shoot length of Roma by 123% and 117%, respectively (Figure 3B). Interestingly, Roma root lengths were significantly lower, by 12% for UQ2077A and 21% for UQ4510An treatments, compared with the control (Figure 3E). The fresh weight of Roma was significantly higher following UQ2077A treatment by 128%, whereas the treatments with UQ4510An and UQ9000N were significantly lower by 7% and 25% compared with the control, respectively (Figure 3H). The Oxheart shoot length was significantly lower by 13% and 12% for UQ2077 and UQ9000N compared with the control, respectively (Figure 3C). The root length of Oxheart was not significantly affected by any of the three isolates compared with the control (Figure 3F). Finally, the fresh weight of Oxheart was significantly higher by 115% for UQ4510AN, and significantly lower by 24% for both UQ2077A and UQ9000N compared with the control (Figure 3I).

### 2.5. Tomato Gene Expression

**Figure 4:**
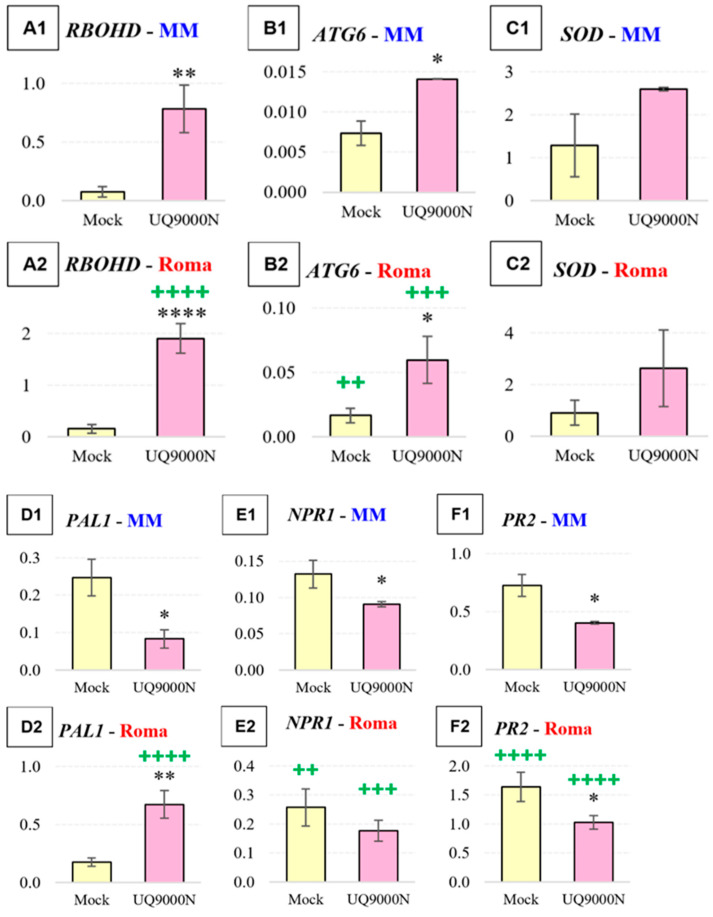

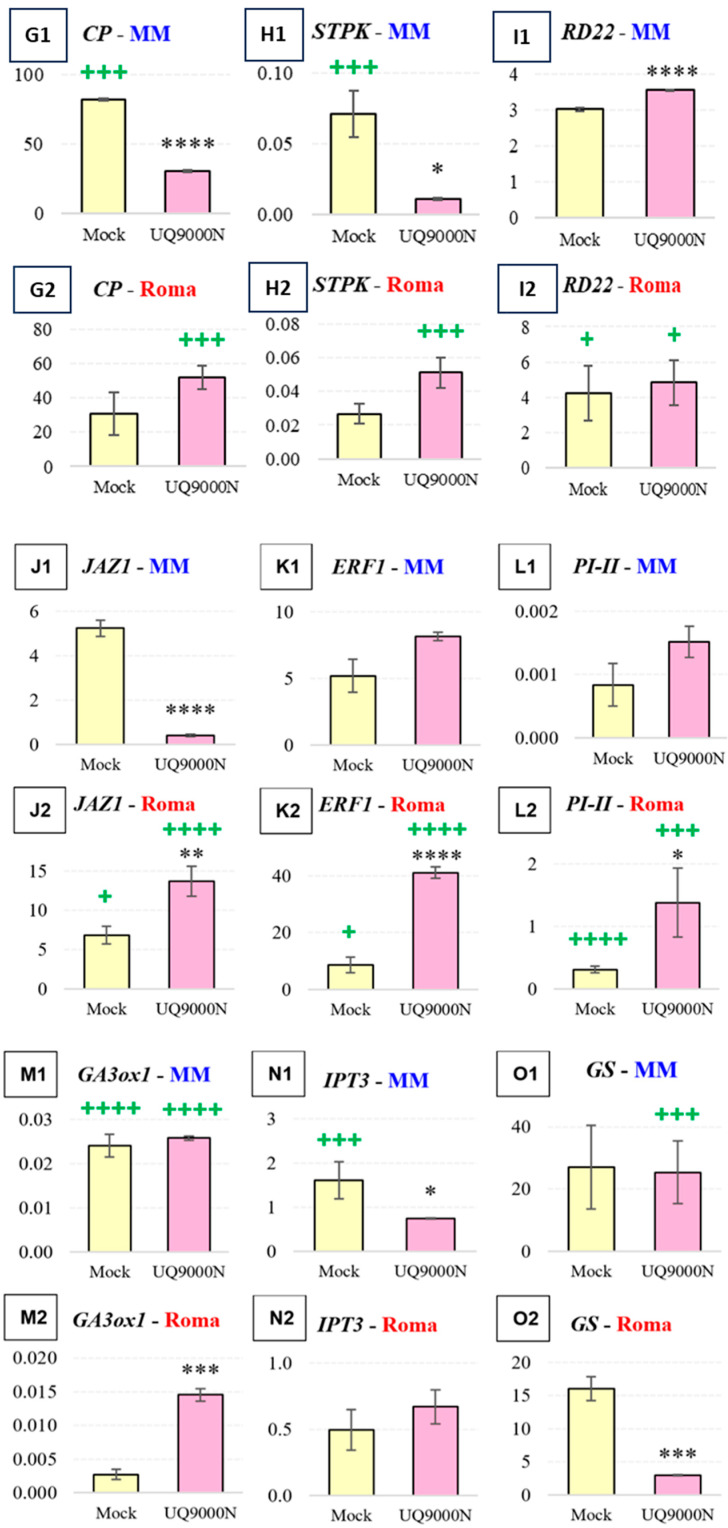
Quantitative real-time RT-PCR analysis of relative gene expression in tomato plants (*S. lycopersicum*; cv. Money Maker and Roma) after pre-treatment with *B. velezensis* UQ9000N at 1 day post inoculation (dpi). Expression values were normalized by the transcript levels of *SlACTIN*. Shown are mean values ± SE (n = 3 biological replicates with 5 plants each) of (**A**–**O**) 15 genes from shoot samples of 4-week-old plants treated with PGPR compared with mock-treated with 1xPBS control plants (Mock). The statistical significance was determined by Student’s *t*-test; asterisks show significant differences to the untreated control plants with * *p* ≤ 0.05, ** *p* ≤ 0.01, *** *p* ≤ 0.001 and **** *p* ≤ 0.0001. In addition, + *p* ≤ 0.05, ++ *p* ≤ 0.01, +++ *p* ≤ 0.001 and ++++ *p* ≤ 0.0001 indicate statistically significant differences for either base or induced levels of genes for equivalent same samples (Mock vs. Mock, 9000 vs. 9000) between the two tomato cultivars.

Gene expression profiling of 15 marker genes (defense and nutrient acquisition) was conducted to investigate whether the cultivar-specific responses to PGPR treatments had a genetic basis by analyzing the contrasting tomato cultivars Money Maker and Roma following treatment with *B. velezensis* isolate UQ9000N. As shown in Figure 4, gene expression after treatment with UQ9000N was significantly (*p* < 0.05) different between the two tested tomato cultivars.

Three marker genes involved in reactive oxygen species (ROS) signaling were examined, including *RBOHD*, *ATG6* and *SOD*. At 24 h post UQ9000N treatment, a significant (*p* < 0.05) increase in *RHOHD* transcripts was observed in both tomato cultivars (11.1-fold in Money Maker and 12.7-fold in Roma) compared with mock-treated control plants (Figure 4A1,A2). Also, the *RBOHD* basal transcript abundance (Mock vs. Mock) was similar in both cultivars, whereas the induced transcript abundance (UQ9000N vs. UQ9000N) was 2.5-fold significantly (*p* < 0.05) higher in the Roma cultivar compared with Money Maker. Similarly, *ATG6* was strongly induced in both cultivars (2-fold in Money Maker and 3-fold in Roma), with higher basal and induced transcript abundances (2.9-fold and 4.3-fold, respectively) in the Roma cultivar (Figure 4B1,B2). On the other hand, *SOD* expression was not significantly (*p* > 0.05) different in UQ9000N-treated samples compared to Mock in both cultivars (Figure 4C1,C2). Also, the *SOD* basal and induced transcript abundances were not significantly (*p* > 0.05) different between the Money Maker and Roma cultivars.

Five marker genes involved in the SA signaling pathway (*PAL1*, *NPR1*, *PR2*, *CP* and *STPK*) were investigated. Following the treatment with UQ9000N, the *PAL1* marker gene was strongly induced (3.5-fold) in the Roma cultivar and significantly downregulated (2.75-fold) in Money Maker (Figure 4D1,D2). The *PAL1* basal transcript levels were not significantly (*p* > 0.05) different between the two cultivars, whereas the induced transcript levels were significantly (*p* < 0.05) higher (8.75-fold) in the Roma cultivar. A significant (*p* < 0.05) decrease (1.4-fold) of *NPR1* transcripts was observed in Money Maker after UQ9000N treatment compared with the control, while the transcript levels were not significantly (*p* > 0.05) different in Roma (Figure 4E1,E2). Both, the *NPR1* basal and induced transcript abundances were significantly (*p* < 0.05) higher (2.3-fold and 2.2-fold, respectively) in the Roma cultivar in comparison with Money Maker. While *PR2* expression was significantly (*p* < 0.05) downregulated in both cultivars (1.75-fold in Money Maker and 1.6-fold in Roma), its basal and induced transcript abundances were higher (2.3-fold and 2.5-fold, respectively) in Roma (Figure 4F1,F2). The transcript levels of *CP* and *STPK* were significantly (*p* < 0.05) downregulated (2.7-fold and 7-fold, respectively) in Money Maker and not significantly (*p* > 0.05) different in Roma compared with control plants (Figure 4G1,G2,H1,H2). Interestingly, the basal transcript abundances for *CP* and *STPK* genes were higher (2.6-fold and 2.3-fold, respectively) in Money Maker, whereas the induced transcript abundance for both genes was higher (1.7-fold and 5-fold, respectively) in Roma. The marker gene used for the ABA signaling pathway (*RD22*) was significantly (*p* < 0.05) upregulated (1.2-fold) in Money Maker after treatment with UQ9000N compared with the control plants, whereas in Roma there was no significant (*p* > 0.05) difference (Figure 4I1,I2). Both the basal and induced *RD22* transcript abundances were higher (1.4-fold and 1.3-fold, respectively) in the Roma cultivar compared with Money Maker.

Three genes involved in the jasmonic acid (JA) and ET pathway were examined, namely *JAZ1, ERF1* and *PI-II*. *JAZ1* was significantly (*p* < 0.05) downregulated (13-fold) in Money Maker after treatment with UQ9000N compared with the control plants, while it was significantly (*p* < 0.05) upregulated (2-fold) in Roma (Figure 4J1,J2). Both the basal and induced *JAZ1* transcript abundances were higher (1.3-fold and 35-fold, respectively) in Roma compared with Money Maker. Significant increases in *ERF1* and *PI-II* transcript levels (4.6-fold and 4.7-fold, respectively) were measured in Roma after treatment with UQ9000N, while transcript abundances of both marker genes were not significantly different to the control plants in Money Maker (Figure 4K1,K2,L1,L2). Similarly, the basal and induced transcript abundances were higher for *ERF1* (1.8-fold and 5.1-fold, respectively) and *PI-II* (375-fold and 933-fold, respectively) in Roma compared with Money Maker.

The marker gene for gibberellic acid (GA) (*GA3ox1*) was strongly induced (5-fold) in the Roma cultivar after the UQ9000N treatment, while in Money Maker it was not significantly (*p* > 0.05) different in comparison with the control plants (Figure 4M1,M2). However, both the basal and induced *GA3ox1* transcript abundances were significantly (*p* < 0.05) higher (6.7-fold and 2-fold) in the Money Maker cultivar compared with Roma. After the UQ9000N treatment, the marker gene *CK* (*IPT3*) was significantly (*p* < 0.05) downregulated (2.3-fold) in the Money Maker cultivar, while in Roma it was not significantly (*p* > 0.05) different in comparison with control plants (Figure 4N1,N2). The basal *IPT3* transcript abundance was significantly (*p* < 0.05) higher (3.2-fold) in Money Maker compared with Roma, while the induced transcript abundance was not significantly (*p* > 0.05) different between the two cultivars.

Finally, the gene encoding GS, a key enzyme for nitrogen metabolism, was significantly (*p* < 0.05) downregulated (5.4-fold) after the UQ9000N treatment in the Roma cultivar, while it was not significantly (*p* > 0.05) different in the Money Maker cultivar compared with control plants (Figure 4O1,O2). Both the basal and induced *GS* transcript abundances were significantly higher (1.7-fold and 8.5-fold) in the Money Maker cultivar compared with Roma.

## 3. Discussion

### 3.1. Functional Properties of the Identified PGPR

Many PGPR genera have been studied and tested as biofertilizer and biopesticide candidates, including *Bacillus*, *Pseudomonas* and *Enterobacter* genera for improving the growth, yield and tolerance against abiotic and biotic stresses of many economically important crops [37,38]. This is in line with the current study that identified the four bacterial isolates as *B. amyloliquefaciens* 33YE, *B. velezensis* UQ9000N, *E. ludwigii* UQ2077A and *P. azotoformans* UQ4510An.

It was found that all four bacterial isolates possess plant growth promotion traits, including production of phytohormone IAA, uptake of nutrients (nitrogen fixation by all four isolates and phosphate solubilization by 33YE and UQ2077An) and production of biofilm (Table 1). *B. amyloliquefaciens* 33YE (tested on two plant species) significantly improved the growth of broccoli cv. Bridge (Figure 1). *B. velezensis* UQ9000N significantly improved growth of all three tested plant species, namely broccoli cv. Bridge, cucumber cv. Lebanese and tomato cv. Money Maker (Figure 1, Figure 2 and Figure 3). *E. ludwigii* UQ2077A (tested on two plant species) improved growth of broccoli cv. Bridge and tomato cv. Money Maker and Roma. *P. azotoformans* UQ4510An (tested on two plant species) significantly improved the growth of cucumber cv. Lebanese and tomato cv. Money Maker and Oxheart (Figure 2 and Figure 3). IAA is one of the most studied plant hormones [39,40] and about 80% of rhizobacteria can produce IAA, which controls various plant growth and developmental processes [40]. Formation of biofilm by PGPR is an important part of plant root colonization, and their activity as biostimulants and biocontrol agents in the rhizosphere microbiome mainly occurs in a biofilm environment [41,42,43]. Many studies have reported that various strains of *B. amyloliquefaciens*, *B. velezensis*, *E. ludwigii* and *P. azotoformans* improve the growth of different crops including tomato (*S. lycopersicum*) and cucumber (*C. sativus*) under growth room, greenhouse and field conditions by fixing N, solubilizing P and K, forming biofilm, producing siderophores, IAA, VOCs (e.g., acetoin, benzaldehyde and 2,3-butanediol) and synthesizing enzymes, including ACC deaminase, phytase, phosphatases and ureases [44,45,46,47,48,49,50,51,52,53,54,55,56,57,58,59].

### 3.2. Role of Plant Genotype for Beneficial PGPR Interactions

Wallenstein [18] hypothesized that there is a genotypic and phenotypic basis for the ability of a plant to support a beneficial microbiome and that this presents a plant trait under selection. Indeed, Wintermans et al. [19] have demonstrated that there is vast natural variation in *Arabidopsis* plant ecotypes that underlies the ability of plants to benefit from a single PGPR (*Pseudomonas simiae* WCS417r). We expected that a similar variation can be expected for different vegetable crop cultivars and that choosing a microbe-friendly genotype can result in improved plant growth. Plant cultivar-specific responses to beneficial PGPR have been shown in various crop plants, including wheat [25,28], maize [29], rice [27,30], hemp [60], and tomato [26,31,32,61,62] in response to PGPR isolates from *Azospirilum* sp., *Bacillus* sp., *Pseudomonas* sp., *Klebsiella pneumoniae* and *Citrobacter freundii*, although their mode of action remains unclear. To this end, the present study focused on the cultivar-specific differential expression of defense and nutrient acquisition genes in the tomato cultivars Money Maker and Roma using *B. velezensis* UQ9000N as an example as there is a good amount of the literature available for this species.

In rice plants, Drogue et al. [27] and Sasaki et al. [30] reported that the host genotype affects the plant’s response to inoculation with PGPR *Azospirillum* sp. isolates. Sasaki et al. [30] concluded that the genotype of the plant, growth stage and management of fertilization (i.e., nitrogen levels) are important factors to consider before using a beneficial microorganism under field conditions. Rozier et al. [29] reported cultivar-specific responses in wheat to *Azospirillum lipoferum* that resulted in the accelerated radicle emergence of one cultivar and a delayed upgrading of seedling defenses. Metabolomic analyses showed increased contents of three substances including ABA and suggested that the germinating wheat seeds of the negatively affected cultivar developed a defense response towards the *A. lipoferum* isolate. This is consistent with the observed defense responses for the Roma tomato variety after inoculation with UQ9000N (Figure 4). Indeed, Roma is considered a variety that displays moderate to strong resistance against a number of plant pathogens, while Money Maker is not [63]. Similarly, Drogue et al. [27] reported cultivar-dependent transcriptional changes in genes related to ethylene (ET) and auxin signaling pathways in rice and concluded that it is important to understand the complex interactions between plant host and PGPR isolates involving growth promotion and defense response.

### 3.3. Role of Host Defense Genes for Beneficial Tomato–B. velezensis UQ9000N Interactions

This study hypothesized that different plant defense responses from microbe-friendly and microbe-hostile genotypes could explain the outcome for plant growth from PGPR–plant interactions. Indeed, it was found that the “microbe-friendly” cultivar Money Maker responded positively (growth promotion) with little or no defense responses, while the “microbe-hostile” cultivar Roma responded negatively (no growth promotion or reduced growth) with strong defense responses. This is consistent with another study where Money Maker was found to respond better than other cultivars for growth promotion and *Salmonella* control [61]. It has been reported that during the initial plant–microbe interactions, the plant host may perceive beneficial microbes (i.e., PGPR) as potential invaders which results in triggered immune responses which are very similar or often identical to those triggered by pests and pathogens [64,65]. However, at the later stages of the interactions, the defense-like responses are repressed through friend versus foe distinction mechanisms, thus enabling successful colonization of host roots by the beneficial microbial symbionts [64,65].

In the present study, it has been observed that the Roma cultivar may perceive the beneficial *B. velezensis* UQ9000N isolate as a pathogen, or it cannot regulate its defense genes as well as the Money Maker cultivar to accommodate and interact with PGPR. The complex network and crosstalk between the phytohormones maintain the balance and allocation of limited resources to either growth or defense responses of the plant [66]. Prolonged upregulation of defense pathways can negatively influence the growth of plants as they divert the resources into defense rather than growth [66,67,68].

#### 3.3.1. ROS Signaling

One of the first plant defense responses is initiated by ROS molecules which at low levels act as secondary messengers during plant growth and development, plant–microbe interactions and responses to abiotic and biotic stress [69,70,71,72]. In the present study, three marker genes involved in ROS signaling were examined, namely *RBOHD*, *ATG6* and *SOD*. *RBOHD* encodes NADPH oxidase, which is involved in ROS production in plants during morphogenesis and development, and it has a primary role during stress response [73,74,75]. ROS-stimulated autophagy is also one of the defensive responses in plants against bacterial pathogens and is involved along with *ATG* genes (including *ATG6*) during the regulation of programmed cell death (PCD [66,76,77,78,79]). Both genes (*RBOHD* and *ATG6*) were significantly upregulated in both cultivars (Roma and Money Maker) following PGPR treatment (Figure 4A,B); however, the induced transcript levels of both genes (and the basal level for *ATG6*) were significantly higher in the Roma cultivar. These results could indicate that in the Money Maker cultivar, UQ9000N was less perceived as a pathogen, whereas in Roma, the higher levels of genes involved in ROS biosynthesis and PCD could lead to oxidative stress and subsequent expression of defense genes involved in SA signaling leading to a hypersensitive response (HR) and PCD [80,81,82,83]. The third ROS marker gene was *SOD*, which encodes superoxide dismutase, an enzyme that catalyzes ROS molecules (e.g., hydrogen peroxide, singlet oxygen, superoxide radical, etc. [81,82,84,85]). Interestingly, this gene was not significantly induced in either of the cultivars, which could indicate that the peak of ROS accumulation was not reached at the examined time point (24 h) [80,86].

#### 3.3.2. SA Signaling

The next five examined marker genes are involved in SA signaling, including *PAL1*, *NPR1*, *PR2*, *CP* and *STPK*. At 24 h after treatment with UQ9000N, *PAL1* was strongly induced in Roma and downregulated in Money Maker (Figure 4D). *PAL1* encodes phenylalanine lyase which catalyzes the first step in the phenylpropanoid pathway, producing hundreds of phenolic compounds (many with defensive capabilities), including SA, and is induced by various pathogens, including *Verticillium dahlia*, in a resistant tomato plant cultivar [82,87,88,89]. These findings suggest that ROS- and *PAL1*-mediated plant defense occurred in the Roma cultivar in response to the UQ9000N treatment.

NPR1 is the main regulator of the SA signaling pathway and involved in the induction of systemic acquired resistance (SAR) through the production of pathogenesis-related proteins, including PR2 (β-1,3-glucanase), for general resistance usually against biotrophic and hemibiotrophic pathogens [90,91,92]. The treatment with UQ9000N resulted in significant *NPR1* suppression in Money Maker which also displayed significantly lower *NPR1*-induced expression levels compared with Roma (Figure 4E). A similar pattern was observed for *PR2*. This indicates that Money Maker suppresses its SA defense signaling pathway during interactions with UQ9000N and may allocate its resources towards growth rather than defense. Two other genes, *CP* and *STPK*, which have been shown to be involved in the SA defense pathway, were also downregulated in Money Maker following UQ9000N treatment. In tomato, SA-induced HR (PCD) has been shown to be associated with the upregulation of *CP*s and they have a role as regulators of defense responses in plants [93,94,95,96]. STPKs are involved in microbe- or pathogen-associated molecular patterns (MAMPs or PAMPs) signal cascades leading to defense responses including PCD [97,98]. Sahu et al. [95] reported that both *STPK PBS1* and *CP TDI-65* were significantly upregulated in tomato plants (tolerant cultivar) during an infection with Tomato leaf curl New Delhi virus (ToLCNDV).

Taken together, all five SA marker genes tested (*PAL1*, *NPR1*, *PR2*, *STPK* and *CP*) showed significantly higher transcript levels in Roma compared with Money Maker following UQ9000N treatment (Figure 4D–H). The tomato Roma cultivar is known to possess enhanced pathogen resistance, which could be conferred through increased basal and induced transcript levels of defense genes, providing stronger basal resistance and enabling faster and stronger responses to pests and pathogens [99,100]. On the other hand, this desirable increased defense capability may have selected against the genetic capacity of this tomato cultivar to interact and host microorganisms in a beneficial manner. Modern plant breeding aimed at improving disease resistance in crops may have selected against traits required for hosting and interacting with some PGPR [33].

#### 3.3.3. JA and ET Signaling

JA is a key component of plant development and responses to abiotic and biotic stresses (in particular necrotrophic pathogens) as well as beneficial plant–microbe interactions (incl. priming/ISR). Its crosstalk with other phytohormones (e.g., GA) is essential during the modulation of plant growth and development [66,101,102]. *JAZ1* encodes a nuclear-localized protein involved in JA signaling which is degraded in response to JA stimulus, and is involved in activation of ISR, while ERF1 is a transcription factor regulated by both ET and JA signaling pathways (usually against necrotrophic pathogens [66,103,104,105,106,107,108,109]. *PI-II* encodes a proteinase inhibitor which has an important role in plant defense, and JA, ET and ABA are involved in regulation of wound-induced PI-II production in tomato and potato plants [110,111,112]. All three JA marker genes (*JAZ1*, *ERF1*, *PI-II*) were significantly upregulated in the Roma cultivar following UQ9000N treatment, and their basal and induced transcript levels were also significantly higher compared with Money Maker (Figure 4J–L). These results suggest that the Roma cultivar perceived the beneficial UQ9000N as a threat and strongly induced the JA defense pathway against this PGPR, which may also explain the lack of growth promotion compared with the PBS-mock treated plants (Figure 3).

The JA pathway plays a dual role in beneficial interactions, with PGPR leading to priming/ISR and defense gene expression. In the first case, plant defense is alerted (put on hold) and is primed for a faster and stronger activation of defense responses but not fully activated at the time, but it appears that in Roma this ability to suppress JA defense signaling and activate the friend versus foe distinction in the presence of PGPR may not be fully functional. Several studies also reported higher basal transcript levels of plant defense genes in resistant tomato plant genotypes compared with susceptible genotypes [113,114].

#### 3.3.4. ABA, GA and CK Phytohormones

*RD22* is a marker gene for ABA signaling which provides tolerance to abiotic stress in plants and is antagonistically regulated to defense pathways [115,116,117]. The mild (1.2-fold) but significant (*p* < 0.0001) upregulation of *RD22* in Money Maker (Figure 4I) was likely a consequence of the internal wiring of the signaling pathways, rather than a sign that these plants had abiotic stress. ABA and JA act antagonistically via MYC2, which is a positive regulator for ABA signaling but a negative regulator for JA signaling [118,119]. As JAZ1 (a negative regulator of MYC2) was strongly suppressed in Money Maker (Figure 4J), JA signaling was suppressed and ABA signaling (as indicated by *RD22* induction) was consequently upregulated (Figure 4I).

The interactions (antagonistic and synergistic) between JA and GA signaling pathways are important mediators between plant defense and growth [66,102,106,120,121,122,123]. GA3ox1 is the key enzyme in the production of bioactive GAs which are involved in the growth and development of plants and biotic stresses (e.g., biotrophic and necrotrophic pathogens) [124,125,126,127,128]. Several studies have shown that the synergistic interactions between GA and JA regulate some developmental events, including trichome development and sesquiterpene synthesis [66,129,130]. A study by Ding et al. [131] showed that GA may have a role in tomato defense against potato purple top phytoplasma infection. Hence, the observed GA induction (as indicated by the strong *GA3ox1* upregulation; Figure 4M) in the Roma cultivar may be another sign for the induced defense response against UQ9000N.

IPT3 is an important enzyme which catalyzes the initial rate-limiting synthesis step of cytokinins (CKs) which are involved in many aspects of plant growth and development, but also in resistance against biotrophic pathogens [67,125,132,133]. The downregulation of *IPT3* in Money Maker (but not in Roma, Figure 4N) following treatment with UQ9000N, may therefore be another indication of the suppression of plant defense in Money Maker to enable beneficial plant–microbe interactions.

#### 3.3.5. Nutrient Acquisition

Glutamine synthetase (GS) is one of the main enzymes in plant metabolism involved in nitrogen assimilation and is essential for growth and development [133,134]. Cai et al. [135] showed that in transgenic rice plants with a co-suppressed *GS2* gene, plants exhibited yellow leaves, reduced plant height and dry weight. In the present study, it was found that *GS* transcript levels following UQ9000N treatment were 8.5 times higher in Money Maker than in Roma (Figure 4O), suggesting that this led to growth promotion in Money Maker, but reduced growth in Roma (Figure 3). The expression of *GS* in Roma (but not in Money Maker) was significantly downregulated after a single treatment with UQ9000N (Figure 4O). This further implies that the “microbe-hostile” Roma cultivar prioritized defense against UQ9000N (significantly higher induced defense gene transcript levels compared with Money Maker; Figure 4) over nitrogen metabolism in the plant (reduced *GS* transcript levels; Figure 4O) and reduced plant growth (Figure 3) compared with Money Maker. These growth–defense trade-offs occur when plants establish a favorable energy balance for the defense response and compensate for induced defense genes by suppressing other metabolic pathways [67,136,137,138].

### 3.4. Should We Restore “Microbe-Friendly” Traits in Crop Breeding Programs?

Modern plant breeding programs typically utilize mono-cropping systems under optimal conditions which lead to a reduction in crop biodiversity and diminish the contribution of the rhizosphere microbiome to plant growth and health [33]. Moreover, they may have selected against genetic traits in plant hosts that are essential for interacting and hosting beneficial microorganisms [33]. Over the past few decades, tomato plant breeding has resulted in a loss in genetic diversity, and it has been suggested that modern breeding programs should focus on reintroducing the desirable traits from wild tomato species [36]. On the other hand, the “microbe-hostile” plant cultivars might have lost some of their ability to recruit and accommodate beneficial PGPR isolates due to modern plant breeding which may have selected against genetic traits in plant hosts that are essential for interacting and hosting beneficial microorganisms [18,33,35].

Results of the present study indicate that the microbe-friendly tomato cultivar Money Maker may be more capable of recruiting and accommodating beneficial PGPR depending on its needs, whether it is for growth promotion or biocontrol of pathogens. On the other hand, the microbe-hostile Roma cultivar, which has been bred to be more resistant to pathogens and diseases [63], might have lost some of its ability to recruit and accommodate beneficial PGPR isolates. Both base and induced transcript levels of defense marker genes were many folds higher in Roma compared with Money Maker (Figure 4), while plant growth and housekeeping metabolism (GS expression) were reduced (Figure 3 and Figure 4). So, either the Roma cultivar perceives the beneficial PGPR isolate as a pathogen, or it cannot regulate its defense genes as well as the Money Maker cultivar to accommodate the PGPR. Hence, the inclusion of the trait “microbe-friendly” in breeding programs would be advisable. To be of practical value, the compatibility of certain PGPR to the soil also needs to be established (Batool et al., 2024). Overall customization of the plant–microbe–soil nexus can be achieved using pot and field trials that screen for plant growth promotion following the inoculation of specific PGPR in certain soils [60].

## 4. Conclusions

This study confirms that the genotype of plant cultivars plays a major role in successful plant-beneficial microbial interactions. Gene expression analysis demonstrated that the compatibility between “microbe-friendly” plant cultivars and PGPR is likely to be manifested by their lower basal defense levels and their ability to suppress defense responses while activating nutrient acquisition genes. In contrast, “microbe-hostile” cultivars may display strong defense responses upon interaction with PGPR. A compatibility to benefit from rhizosphere microbes should be considered an important trait when developing new crop cultivars with increased yield and resilience.

## 5. Materials and Methods

### 5.1. Bacterial Cultivation and Inoculum Preparation

*Bacillus amyloliquefaciens* 33YE, *Bacillus velezensis* UQ9000N, *Enterobacter ludwigii* UQ2077A and *Pseudomonas azotoformans* UQ4510An were isolated from clay-rich soil populated by oleander and longan tree roots collected from Tennyson, Queensland, Australia (GPS coordinates 27°31′37.0” S 152°59′51.7” E; [5]). The isolates were pre-cultured from −80 °C glycerol stocks and grown in YEP (yeast extract peptone) broth (L-1: 10 g bactopeptone, 10 g yeast extract, 5 g NaCl) overnight on a flat shaker incubator at 28 °C in 50 mL Falcon tubes with 25 mL of medium in each tube (100 rpm). After 24 h, 1 mL aliquots obtained from these pre-cultures was added into a fresh YEP broth medium in 50 mL Falcon tubes and again incubated overnight under the same conditions. Then, the suspension was diluted with YEP broth (for broccoli) or phosphate-buffered saline (PBS) (for cucumber and tomato) to a final optical density at 600 nm (OD600) of 0.1, corresponding to 10^6^ colony forming units (CFUs) mL^−1^ for *Bacillus* spp. and 10^7^ CFU mL^−1^ for *E. ludwigii* and *P. azotoformans*. This served as the main inoculum culture of all experiments. YEP-only or PBS-only acted as mock-treated controls.

### 5.2. Bacterial DNA Isolation and Identification via 16S rDNA Gene Amplicon Sequencing

A volume of 20 µL of overnight bacterial culture from YEP broth was used to inoculate YEP plates to obtain single colonies. After 10 h growth, single colonies were extracted with a 1 µL loop and lysed at 95 °C in nuclease-free water for 10 min. The lysates were centrifuged at 12,000× *g* for 3 min twice to remove extracellular matrix and media. An amount of 25 µL PCR reactions was then set up with sample supernatants with commercial master mix MangoMixTM (Bioline, London, UK). The primers used in this study were universal primers 27F 5′-AGAGTTTGATCMTGGCTCAG-3′ and 1492R 5′-TACGGYTACCTTGTTACGACT-3′). The PCR amplification was confirmed with a 1% (*w*/*v*) agarose gel electrophoresis, and the DNA concentration was confirmed using a Qubit Fluorometer (Thermo Scientific, Waltham, MA, USA) and quantified with a Nanodrop Spectrophotometer (Thermo Scientific). The amplified PCR products were submitted to the Australian Genome Research Facility Ltd. for two-directional Sanger sequencing.

### 5.3. Phylogeny Analysis

Nucleotide sequences of the 16S rRNA gene amplicons of each of the three newly isolated strains, namely *B. velezensis* UQ9000N, *E. ludwigii* UQ2077A and *P. azotoformans* UQ4510An, were compared to known sequences in the National Library of Medicine (collection consists of GenBank + EMBL + DDBJ + PDB + RefSeq sequences) database using BLASTN search to determine the taxa of the isolates [139]. A phylogenic tree of each of the three isolates was made using the Molecular Evolutionary Genetics Analysis (MEGA) software version 11.0 after multiple alignments of data by CLUSTAL W [140,141]. The relationships between each isolate and other known sequences were analyzed using the neighbor-joining method with 1000 bootstrap replicates [141,142,143,144].

### 5.4. Plant Growth Promotion Traits

#### 5.4.1. Indoleacetic Acid Production

Production of indole-3-acetic acid (IAA) by the four PGPR isolates was measured using the previously described method with some modifications [145,146]. A volume of 0.5 mL of overnight bacterial culture was centrifuged at 11,000× *g* for 5 min and the pellet was resuspended in 1 mL of Luria–Bertani (LB) broth supplemented with sterile 800 µg mL^−1^ L-tryptophan and incubated at 25 °C in the dark for 4 days. LB broth comprised (L^−1^), including tryptone 10 g, yeast 5 g, NaCl 5 g and distilled water up to 1 L. After that, the bacterial culture was centrifuged at 11,000× *g* for 10 min. Subsequently, 0.5 mL of the supernatant was mixed with 0.5 mL of Salkowski’s reagent (distilled water 49 mL, 70% perchloric acid 49 mL and 0.5 M FeCl_3_ solution 2 mL). After a 30 min incubation in the dark at room temperature, the before absorbance was measured at 530 nm. An IAA standard curve from 1 to 20 mg mL^−1^ was prepared to calculate the concentration of IAA produced by each of the four PGPR isolates. Each isolate was tested in nine replicates.

#### 5.4.2. Biofilm Production

The biofilm production by the four PGPR isolates was determined using the assay by Erriu et al. [147]. Diluted aliquots (1:100) of 100 µL of overnight culture of each isolate were transferred into a 96-well plate and incubated at 25 °C for 24 h. Then, 10 µL of crystal violet dye 0.1% (*w*/*v*) was added into each well and incubated in the dark at room temperature for 15 min. Each well was thoroughly rinsed with distilled water to remove any unattached planktonic cells and remaining dye. Then, 100 µL of 95% (*v*/*v*) ethanol was added to solubilize the stained dye of the biofilm cells, and the absorbance was measured at 600 nm. The background absorbance was determined using uninoculated medium as a control. Each isolate was tested in nine replicates.

#### 5.4.3. Nitrogen Fixation

The nitrogen fixation trait of the four PGPR isolates was tested using the nitrogen-free bromothymol blue (NFB) assay [146,148,149]. Briefly, 10 µL from each PGPR isolate overnight culture at OD600 of 0.1 was transferred onto NFB agar and incubated at 25 °C in the dark for 4 days. The NFB agar comprised (L^−1^) malate 5 g, agar 15 g, KOH 4 g, K_2_HPO_4_ 500 mg, FeSO_4_.7H_2_O 50 mg, NaCl 20 mg, CaCl_2_ 10 mg, MgSO_4_.7H_2_O 10 mg, MnSO_4_.7H_2_O 10 mg, Na_2_MoO_4_ 2 mg, 0.5% bromothymol blue alcoholic solution 2 mL (bromothymol blue powder 0.5 g in 50 mL of 95% ethanol and then 50 mL of distilled water) and distilled water up to 1 L and pH adjusted to 6.8. Each experiment had three technical replicates per sample and was repeated three times.

#### 5.4.4. Phosphorus Solubilization

The calcium phosphate solubilization ability of the four PGPR isolates was tested using Pikovkaya’s medium assay [146,150]. Briefly, a volume of 10 µL of each PGPR isolate overnight culture at OD600 of 0.1 was transferred onto Pikovkaya’s medium agar plates and incubated at 25 °C in the dark for 3 days. Pikovkaya’s medium agar comprised (L^−1^) agar 15 g, dextrose 10 g, Ca_3_(PO_4_)_2_ 5 g, yeast extract 500 mg, (NH_4_)_2_SO_4_ 500 mg, MgSO_4_ 100 g, MnSO_4_ 0.1 mg, FeSO_4_ 0.1 mg, and distilled water up to 1 L. Subsequently, the solubilization zone was calculated as bacterial colony diameter (mm) and subtracted from the halo zone diameter (mm). Finally, the Pi solubilizing index (PSI) was calculated as halo zone diameter (mm) + bacterial colony diameter (mm)/bacterial colony diameter (mm) [151]. Each experiment had three technical replicates per sample and was repeated three times.

### 5.5. Plant Treatments

Tested plants included broccoli (*Brassica oleracea* var. *italica*; cv. Solitaire and Bridge), cucumber (Cucumis sativus; cv. Lebanese and Marketmore) and tomato (*Solanum lycopersicum* L.; cv. Money Maker, Oxheart and Roma). Different plant species had different growth conditions as described in the following section. All harvested plant samples were stored at −80 °C until further genetic analysis. All pot trials were conducted in double door Conviron growth cabinets with a light intensity of 100 μmol photons m^−2^ s^−1^ (white, fluorescent lamps), day/night cycles, temperature and humidity as described below.

#### 5.5.1. Broccoli Plant Cultivation

Seeds of two broccoli cultivars, Solitaire and Bridge, were provided by QDAF (Queensland Department of Agriculture and Fisheries, Australia). The broccoli was grown in a growth cabinet over 7 weeks with day/night temperatures at 24/21 °C, day/night cycles 10/14 h and relative air humidity 60–65%. The broccoli was inoculated with strains UQ2077A, UQ9000N and 33YE associated with control plants that were mock-treated with the PBS buffer only. Initially, the broccoli seeds were soaked in bacterial suspension at OD600 of 0.1 or PBS buffer for 1 h and then sowed in UQ23 potting mix composed of composted pine bark (up to 5 mm; 70%), cocoa peat (30%) and mineral fertilizer. Follow-up root inoculations occurred at week 3 via pipetting (4 mL per plant), as repeat inoculations had previously shown benefits with other vegetable crops [5,60]. Each group had 25–30 individual plants (one plant per pot). Plant parameters were measured for both cultivars at harvesting.

#### 5.5.2. Tomato and Cucumber Cultivation

Tomato (cv. Money Maker, Oxheart and Roma) and cucumber (cv. Lebanese and Marketmore) plants were used for the experiments. Initially, seeds were surface-sterilized with 70% ethanol for 5 min, followed by soaking in 1% sodium hypochlorite for a further 3 min, and then rinsed with sterilized distilled water five times. Subsequently, seeds were placed onto sterilized filter paper moistened with sterile water for 5 and 2 days for the tomato and cucumber seeds to germinate, respectively. Then, the germinated tomato and cucumber seeds were transferred to 55 × 65 mm 30-cell trays and 85 × 100 mm square pots, respectively, and filled with moist UQ23 potting mix, one seedling per pot. Plants were watered by pouring deionized water into the tray to be absorbed by the soil every 2–3 days. The plant seedlings were kept in a growth cabinet at 12 h of light, 25 °C day/19 °C night and 70% humidity. After 2 weeks of growth, the plants were inoculated with bacterial formulations by adding 5 mL of bacteria per plant at the base of the stem. After one more week of growth (3-week-old seedlings), tomato and cucumber plants were harvested and phenotypic measurements were recorded, including plant shoot and root length and fresh weight.

### 5.6. Tomato Defense Gene Expression Analysis

To further investigate how the plant cultivar genotype dictates the difference in response to PGPR isolates, real-time quantitative reverse transcriptase PCR (qRT-PCR) was used to measure the expression of defense marker gene expression in tomato plants. One PGPR isolate was chosen, namely *B. velezensis* UQ9000N, and two tomato cultivars were chosen, Money Maker and Roma, as they reacted positively and negatively, respectively, to the treatment with this bacterial isolate. Total RNA isolations were performed on frozen tomato shoot samples with a Maxwell RSC Plant RNA Kit (PROMEGA) following the manufacturer’s instructions. The concentration and purity of the obtained RNA samples were measured using a Nanodrop Spectrophotometer (Thermo Scientific). The cDNA was generated by reverse transcription using the Tetro cDNA synthesis kit (Bioline), following the manufacturer’s instructions. The reactions used 12 µL of RNA samples at a concentration of 10 ng μL^−1^ (total amount ~120 ng) in a 20 µL reaction using both random hexamers and oligo dT primers. Real-time qPCR was performed on the QuantStudio6 384 well plate Real-Time PCR System (Applied Biosystems, Foster City, California, USA). The primers used for this experiment are shown in Table 2.

Each reaction mixture with a volume of 10 μL contained 4 μL of sample DNA (~10 ng μL^−1^), 5 μL of SYBR green master mix, and 1 μL of mixed forward and reverse primers (3 µM). *SlACTIN* was used as the housekeeping gene for normalization. Thermal cycling conditions were set as follows: (1) the heat activation step with 1 cycle of 95 °C for 2 min, then (2) the amplification step with 40 cycles of 95 °C for 10 s and 60 °C for 20 s, followed by (3) the melt curve analysis step with 1 cycle of 95 °C for 15 s, 60 °C for 1 min and 95 °C for 15 s. Relative expression of each target gene was investigated using three biological replicates (five plants each) with three technical replicates. Data analysis was performed with QuantStudio™ Real-Time PCR Software v1.1 (Applied Biosystems). Relative expression (n-fold) of the normalized target genes in both treatments was determined as proposed by Pfaffl [158].

### 5.7. Statistical Analysis

For comparisons between treatments, significant changes (*p* < 0.05) were determined based on Student’s *t*-test for pairwise comparisons or ANOVA F test followed by Tukey’s HSD test using JMP software. Graphs were prepared using Microsoft Excel.

## Figures and Tables

**Table 1:** Summary of plant beneficial traits exhibited by the four soil bacterial isolates used.

PGPR Isolate	16S rDNA Sequencing Alignment	IAA Production	Biofilm Production	Nitrogen Fixation	Phosphate Solubilization
33YE	*Bacillus* *amyloliquefaciens*	**√**	**√**	**√**	**√**
UQ2077A	*Enterobacter* *ludwigii*	**√**	**√**	**√**	**√**
UQ4510An	*Pseudomonas* *azotoformans*	**√**	**√**	**√**	**-**
UQ9000N	*Bacillus velezensis*	**√**	**√**	**√**	**-**

The tick (√) indicates positive results, and minus indicates negative results.

**Table 2:** Primers used for qRT-PCR.

Target Gene	Forward Primer (5’-3’)	Reverse Primer (5’-3’)	Reference
*SlRBOHD* *SlATG6* *SlSOD* *SlPAL1* *SlNPR1* *SlPR2* *SlCP* *SlSTPK* *SlRD22* *SlJAZ1* *SlERF1* *SlPI-II* *SlGA3ox1* *SlIPT2* *SlGS* *SlACTIN*	TCAGGTCAAGCATCAAAGCCGTT CCCATGCAGTCAAACAATTC CAAGATGATGATGGTCCAAC CATTGTACAGGTTGGTGAGAG TGTGGGAAAGATAGCAGCACG TTTCGATGCCCTTGTGGATTC TCCGAAGGCCCCAATAGG TGCATTGCAAACAGCAACAA ACGTGGCGTTATTTTTTCCTG TTCCCTCAAGGTGGAATGAAGGCT AGACTTGGGAGTTGAATTA CTTCTTCCAACTTCCTTTG GAATCCCATGCATGGACATCAT CCTTCTTGCACAAAGTTGCT CGCCGCCCAGCTTCAAACAT AGGCAGGATTTGCTGGTGATGATGCT	TGGTGAAACCGCAGCACAGT CCCTCATGCATTCAAGACAC CTCCATGTGTCAATTTATTCGG CATCTCTTGAGACACTCCA GTCCACACAAAACACACACATC GGCCAACCACTTTCCGATAC CACTGGGAGTGAAGGCAATGA CCAAGAGATCCTTCACCAATGAG ATCTCCGGCATCTTCTCTGA TCCGAAACTCGGAACCACCAAATC TACATTGCGATCTTGATTA TGTTTTCCTTCGCACATC TGTTATCGAGGTCGATCACTGG TGAGGTTATTGAATATTAGCAAATA CCTCAAGGGTTGGCTCCCACA ATACGCATCCTTCTGTCCCATTCCGA	[75] [75] [152] [153] [154] [135] [99] [99] This study [155] [153] [111] [156] [133] [157] This study

## Data Availability

The data presented in this study are available in this article.

## Supplementary Materials

The following supporting information can be downloaded at: https://www.mdpi.com/article/10.3390/plants13213065/s1, Figure S1. Phylogenetic trees based on 16S rRNA gene sequences.
