# Supplementary material for: Microbe-Friendly Plants Enable Beneficial Interactions with Soil Rhizosphere Bacteria by Lowering Their Defense Responses
Source: Plants (Basel). 2024 Oct 31;13(21):3065. doi: 10.3390/plants13213065 (PMC11548416; doi:10.3390/plants13213065)
Supplement: Supplementary file 1 [file plants-13-03065-s001.zip › plants-3246605-supplementary.pdf]

Figure S1

(A) *Bacillus velezensis* UQ9000N phylogenetic tree

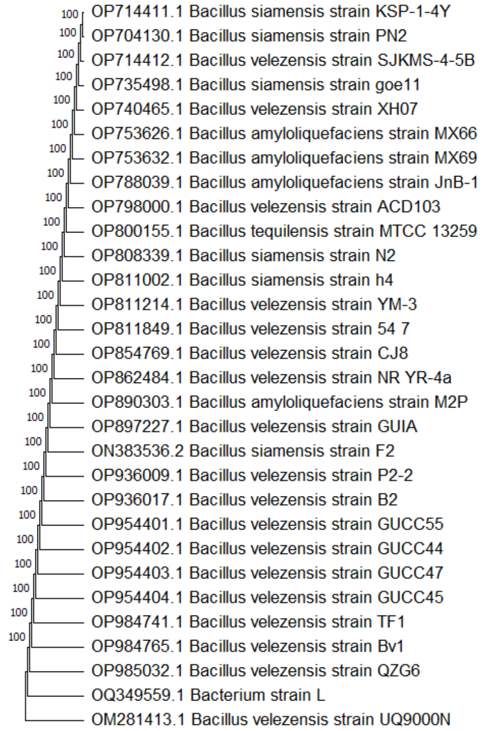

Figure S1. Phylogenetic trees based on 16S rRNA gene sequences. The relationships between each of the (A) *Bacillus velezensis* UQ9000N, (B) *Enterobacter ludwigii* UQ2077A and (C) *Pseudomonas azotoformans* UQ4510An and other known related strains/species derived from NCBI accessions. The tree was inferred using the neighbor-joining method and MEGA 11.0 software with 1000 bootstrap replicates (bootstrap values are shown next to nodes).

(B) *Enterobacter ludwigii* UQ2077A

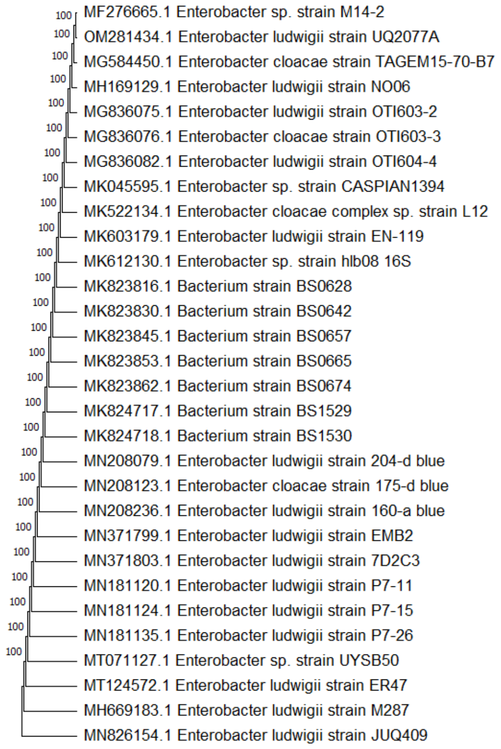

(C) *Pseudomonas azotoformans* UQ4510An

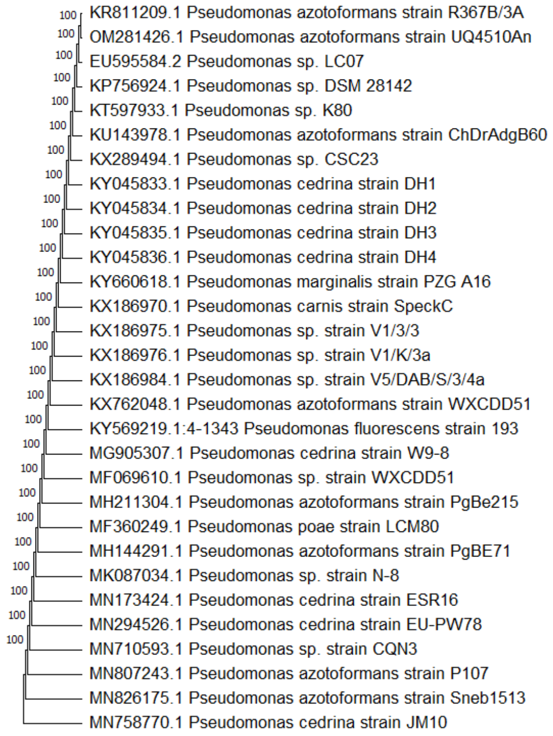

Figure S1. Continued.
